# Overexpressing the novel autocrine/endocrine adipokine WISP2 induces hyperplasia of the heart, white and brown adipose tissues and prevents insulin resistance

**DOI:** 10.1038/srep43515

**Published:** 2017-02-27

**Authors:** John R. Grünberg, Jenny M. Hoffmann, Shahram Hedjazifar, Annika Nerstedt, Lachmi Jenndahl, Johannes Elvin, John Castellot, Lan Wei, Sofia Movérare-Skrtic, Claes Ohlsson, Louise Mannerås Holm, Fredrik Bäckhed, Ismail Syed, Fatima Bosch, Alan Saghatelian, Barbara B. Kahn, Ann Hammarstedt, Ulf Smith

**Affiliations:** 1 The Lundberg Laboratory for Diabetes Research, Departments of Molecular and Clinical Medicine, Institute of Medicine, Sahlgrenska Academy at the University of Gothenburg, Gothenburg, Sweden; 2 Department of Integrative Physiology and Pathobiology, Tufts University School of Medicine, Boston, Massachusetts, USA; 3 Massachussetts General Hospital, Boston, Massachusetts, USA; 4 Centre for Bone and Arthritis Research, Departments of Molecular and Clinical Medicine, Institute of Medicine, Sahlgrenska Academy at the University of Gothenburg, Gothenburg, Sweden; 5 The Wallenberg Laboratory, Departments of Molecular and Clinical Medicine, Institute of Medicine, Sahlgrenska Academy at the University of Gothenburg, Gothenburg, Sweden; 6 Novo Nordisk Foundation Center for Basic Metabolic Research, Section for Metabolic Receptology and Enteroendocrinology, Faculty of Health Sciences, University of Copenhagen, Copenhagen, Denmark; 7 Department of Medicine, Beth Israel Deaconess and Harvard Medical School, Boston, Massachusetts, USA; 8 Center of Animal Biotechnology and Gene Therapy, Universitat Autònoma de Barcelona, Bellaterra, Spain; 9 Salk Institute for Biological Studies, La Jolla, California, USA

## Abstract

WISP2 is a novel adipokine, most highly expressed in the adipose tissue and primarily in undifferentiated mesenchymal cells. As a secreted protein, it is an autocrine/paracrine activator of canonical WNT signaling and, as an intracellular protein, it helps to maintain precursor cells undifferentiated. To examine effects of increased WISP2 *in vivo*, we generated an aP2-WISP2 transgenic (Tg) mouse. These mice had increased serum levels of WISP2, increased lean body mass and whole body energy expenditure, hyperplastic brown/white adipose tissues and larger hyperplastic hearts. Obese Tg mice remained insulin sensitive, had increased glucose uptake by adipose cells and skeletal muscle *in vivo* and *ex vivo*, increased GLUT4, increased ChREBP and markers of adipose tissue lipogenesis. Serum levels of the novel fatty acid esters of hydroxy fatty acids (FAHFAs) were increased and transplantation of Tg adipose tissue improved glucose tolerance in recipient mice supporting a role of secreted FAHFAs. The growth-promoting effect of WISP2 was shown by increased BrdU incorporation *in vivo* and Tg serum increased mesenchymal precursor cell proliferation *in vitro*. In contrast to conventional canonical WNT ligands, WISP2 expression was inhibited by BMP4 thereby allowing normal induction of adipogenesis. WISP2 is a novel secreted regulator of mesenchymal tissue cellularity.

Obesity is the major driving force of the current global Type 2 Diabetes (T2D) epidemic, and the expanded hypertrophic adipose tissue has a central role in this by driving insulin resistance^1^,^2^.

Adipose tissue expansion can, in principle, be induced by hyperplasia with the recruitment of precursor cells into the adipogenic lineage or by hypertrophy of pre-existing adipocytes. Hypertrophic obesity leads to adipose tissue dysfunction, local and systemic inflammation, insulin resistance and tissue fibrosis while hyperplastic obesity is protective^1^,^2^,^3^,^4^.

This protective effect of a hyperplastic adipose tissue was clearly demonstrated in a mouse model overexpressing adiponectin in the adipose tissue^5^. These mice became extremely obese but had hyperplastic tissue and remained metabolically perfectly normal. Similarly, overexpressing Glucose transporter type 4 (GLUT4; also known as SLC2A4) in the adipose tissue induced obesity but with hyperplastic adipose tissue and enhanced glucose tolerance^6^.

Obesity is not only associated with the expanded adipose tissue but also other mesenchymal tissues are enlarged such as the heart^7^, lean body mass and bone density^8^. If these changes are secondary to the increased body weight itself and/or mediated through paracrine/endocrine factors and if the expanded adipose tissue may play a role in this are unknown.

Recently, we identified WNT1-inducible signaling pathway protein 2 (WISP2/CCN5) as a novel secreted adipokine which was increased in obesity and insulin resistance in the subcutaneous adipose tissue in man^9^. Studies of the secretome of human adipose tissue identified WISP2 as the protein with the largest difference in secretion between obese and lean individuals^10^. Furthermore, tissue expression profile in Supplementary Fig. 1a shows that it is most highly expressed in the adipose tissue of all tissues examined.

WISP2 is a multidomain and multifunctional protein with apparent cell-specific effects. In the adipose tissue, it is primarily expressed in undifferentiated mesenchymal precursor (stem)cells and preadipocytes. It is also highly expressed in certain cancer cells where it is a well-established tumor suppressor antagonizing the effect of TGFβ and its induction of epithelial-mesenchymal cell (EMC) transformation^11^. Furthermore, it was recently shown that overexpressing WISP2 in the heart of mice was anti-fibrotic, reducing TGFβ-induced myofibroblast formation and preventing the development of fibrotic heart failure^12^. Importantly, WISP2 protein was also reduced in human fibrotic heart failure^12^.

WISP2 is both an intracellular and secreted protein in many cells including adipose precursor cells^9^,^13^. As an intracellular protein, we found WISP2 to be an important regulator of the effect of BMP4 in committing adipose mesenchymal precursor cells into the adipogenic lineage^9^. WISP2 retains zinc finger protein 423 (ZNF423), a bone morphogenetic protein (BMP)-regulated transcriptional activator of peroxisome proliferator-activated receptor γ (PPARγ)^14^, in the cytosol preventing its nuclear entry^9^. BMP4 is a key regulator of mesenchymal precursor cell adipogenic commitment^2^,^3^,^15^ and BMP4 dissociates the ZNF423/WISP2 complex through SMAD activation and allows ZNF423 to enter the nucleus and induce PPARγ^9^.

WISP2 is also an important regulator of adipose precursor cell growth and differentiation as a secreted protein. Conditionally silencing WISP2 prevented growth of 3T3-L1 cells and initiated spontaneous differentiation of the cells. Furthermore, we found WISP2 to increase the phosphorylation of the LRP 5/6 co-receptor, increasing nuclear β-catenin accumulation and activating the TCF receptor, i.e.; it is an autocrine/paracrine growth factor and activator of the canonical WNT signaling pathway in mesenchymal precursor cells^16^, albeit with distinct differences from conventional canonical WNT ligands^16^.

These findings make WISP2 an interesting growth factor for mesenchymal precursor cells but an *in vivo* model is required to examine its biological effects. We here characterize the effect of WISP2 in lean and obese animals by expressing it in transgenic (Tg) mice under an aP2 promoter and feeding the mice control (LFD) or High Fat Diet (HFD) for 17 weeks.

## Results

WISP2 Tg mice were viable, fertile and appeared normal by gross inspection. We followed them for up to 52 weeks, and they maintained the phenotype described here without any evidence of organ- or behavioral abnormalities or tumors.

**Figure 1:**
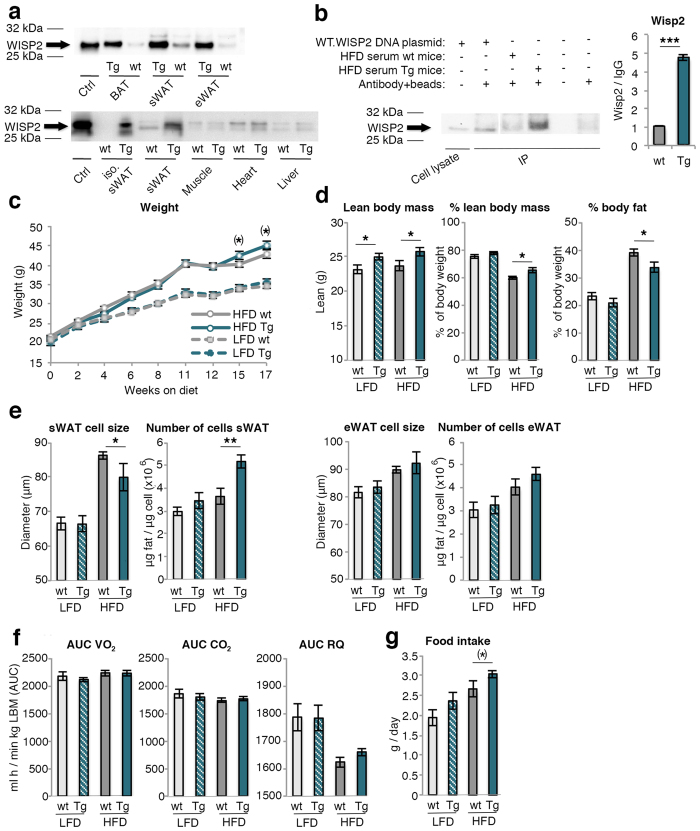
Characterization of WISP2 over-expression, effects on body weight, body composition, adipose cell size and number, food intake and energy expenditure. (**a**) Upper blot shows WISP2 protein expression in adipose tissues from wt and Tg mice; BAT, sWAT and eWAT. Lower blot showsWISP2 protein from isolated mature sWAT adipose cells, whole tissue sWAT, muscle (gastrocnemius), heart and liver from wt and Tg mice. WT.WISP2 DNA plasmid expressed in NIH 3T3 cells was used as a positive control (ctrl). Full-length blots are presented in Supplementary Fig. 7a. (**b**) Wisp2 protein in serum from wt and Tg mice on HFD and quantification normalized to the unspecific band of Ig G (n = 4/group). WT.WISP2 DNA plasmid expressed in NIH 3T3 cells was used as a positive control, antibody + beads was used as negative control. Full-length blots and additional serum samples are presented in Supplementary Fig. 7b. (**c**) Body weights (n = 27–40/group) and (**d**) body composition assessed by DEXA (n = 12–18/group). (**e**) Adipose cell size and number of cells in sWAT and eWAT (n = 11–13/group) and (**f**) energy expenditure data normalized to lean body mass are displayed as area under the curve (AUC) after 15 weeks on diets (n = 8/group). (**g**) Food intake normalized to body weight (n = 5–9/group). The experimental data are presented as means ± SEM. 2-way ANOVA was used to compare ≤4 groups; otherwise Student’s t-test was used. ***p < 0.001, **p < 0.01, *p < 0.05, (*)p < 0.1.

WISP2 protein is expressed in the adipose tissue of wt mice and increased in Tg mice (Fig. 1a-top). This increase was, as expected, mainly seen in mature and differentiated adipose cells (Fig. 1a-below) while no difference was seen between wt and Tg mice in WISP2 expression in skeletal muscle, heart and liver (Fig. 1a-below). Similarly, there was no difference in expression between wt and Tg mice in undifferentiated adipose tissue stromal vascular cells, which includes endothelial cells (Relative quantification (RQ) 1.0 vs. 1.7, NS) or in peritoneal macrophages (Supplementary Fig. 1b). This was tested because the aP2 promoter has been shown to be able to target macrophages and weakly endothelial cells^17^ but we saw no such effects. We also examined expression profile in the macrophages and there was no difference in either the M1 or the M2 phenotypes between the wt and Tg mice (Supplementary Fig. 1c). Thus, we conclude that the adipose tissue and the differentiated adipose cells were the predominant sites of increased protein expression in Tg mice. However, in spite of this quite extensive examination of ectopic WISP2 gene expression, we can not completely exclude other sites not examined such as the brain.

WISP2 levels were also markedly increased in serum of Tg animals showing that it is a secreted and circulating protein released by the adipose tissue (Fig. 1b and Supplementary Fig. 7b).

### Body weight and composition

At the age of 6 weeks, when the LFD and HFD diets were initiated, the mean body weights of Tg (20.7 ± 0.4 g) and wildtype (wt) littermates were similar (21.3 ± 0.3 g). Both LFD and HFD increased body weights in wt and Tg animals to a similar extent although the Tg mice tended to weigh slightly more (Fig. 1c) and this was also seen in a separate cohort followed for 52 weeks on chow diet (Supplementary Fig. 2a). The variability in growth in Fig. 1c is a consequence of the phenotyping procedures performed from week 11 onwards.

**Figure 2:**
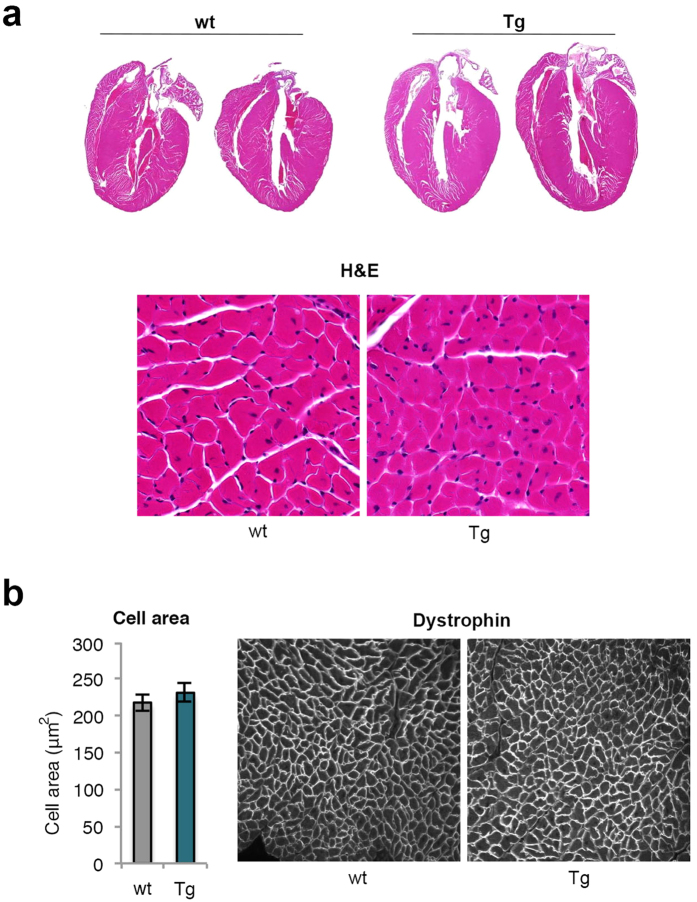
Increased heart size and cell number but not cell size in transgenic mice. (**a**) Representative heart sections from 23 weeks old wt and Tg mice on HFD and the left ventricular wall (20x magnification) visualized with Hematoxylin staining. (**b**) Quantification of cell area (n = 4/group) of the left ventricular wall (20x magnification) stained with anti-dystrophin. The experimental data are presented as means ± SEM. Student’s t-test was used.

Body composition analyses showed that Tg mice on HFD had significantly increased % lean body mass (LBM) and lower % body fat (BF). Also total LBM tended to be increased in both the LFD and HFD groups (Fig. 1d), mainly attributable to increased weights of skeletal muscles, brown adipose tissue (BAT) and the heart; the latter increased by 20–30% (Table 1). Importantly, the increased heart weights were not due to hypertrophy of the cells but to an increased number/hyperplasia of cardiomyocytes (Fig. 2a,b). Pooled muscle and heart weights were significantly increased by around 15–20% (Table 1) but no significant differences were seen in femur and tibia bone parameters either in bone density or in bone length (Supplementary Table 1).

BAT weights were about 2-fold higher in LFD Tg animals and about 50% higher in HFD (Table 1) and eWAT and retroperitoneal WAT (rWAT) weights were increased in both HFD and LFD Tg mice (Table 1).

In spite of similar total sWAT weight, HFD Tg mice had smaller subcutaneous adipocytes but an increased number per tissue weight compared to wt (Fig. 1e). This hyperplasia was also confirmed by adipocyte cell size distribution and a similar trend was seen in eWAT (Fig. 1e). Histological analysis also revealed a hyperplastic BAT with fewer and smaller lipid droplets in HFD Tg than in wt mice but there was no difference in UCP1 activation either by *Ucp1* mRNA (Table 2), staining or protein quantification (Supplementary Fig. 3a,b).

Thus, WISP2 Tg mice on HFD showed increased muscle mass, increased mass and hypercellularity of the heart, BAT and the WAT regions which typically expand when overfeeding mature mice^18^,^19^.

### Food intake, energy expenditure and activity patterns

Oxygen consumption was significantly increased in the HFD Tg compared to wt mice when expressed per total body weight (Supplementary Fig. 2b). However, this was entirely due to the increased lean body mass since expressing energy expenditure per kg lean body mass removed the differences (Fig. 1f). Similarly, respiratory quotient was unchanged (Fig. 1f) and there was also no difference in activity patterns between the Tg and wt mice (Supplementary Fig. 2c). The increased whole-body energy expenditure was compensated for by increased food intake in Tg mice (Fig. 1g).

### Glucose and insulin homeostasis in wt and HFD Tg fed mice

**Figure 3:**
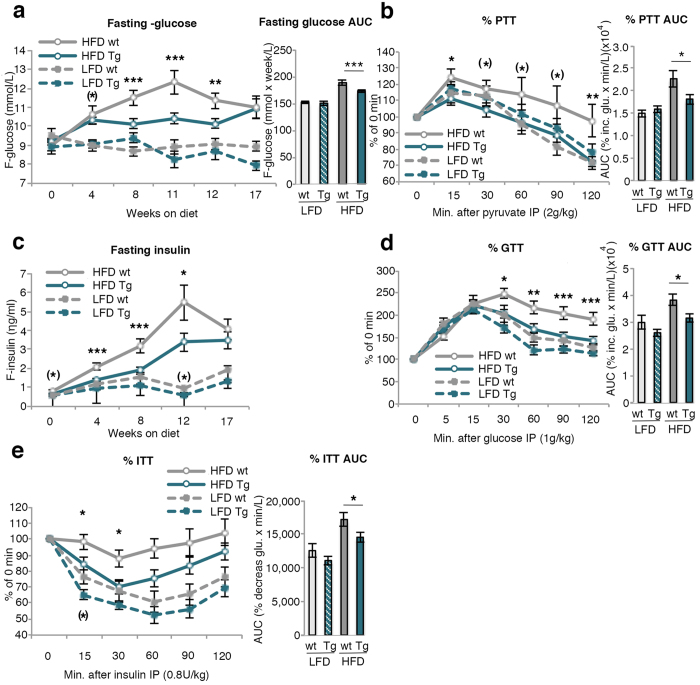
Characterization of glucose and insulin homeostasis. (**a**) Fasting glucose during a 17 week period and area under the curve (AUC) was calculated (n = 24–40/group) (**b**) intraperitoneal pyruvate tolerance test (PTT) at 12 weeks on respective diet, calculated as percentage of the fasting value at time point 0. AUC was calculated from the PTT curve (n = 11–21/group). (**c**) Fasting insulin during a 17 week period (n = 24–39/group) (**d**) Intraperitoneal glucose tolerance test (GTT) and intraperitoneal insulin tolerance test (ITT) (**e**) at 11/12 weeks on diet, calculated as percentage of the fasting value at time point 0. AUC was calculated from the GTT and ITT curve (n = 11–18/group). The experimental data are presented as means ± SEM. ANOVA was used to compare ≤4 groups. ***p < 0.001, **p < 0.01, *p < 0.05, (*)p < 0.1.

Neither glucose (9.2 and 9.1 mM in wt and Tg animals, respectively) nor insulin (0.68 and 0.61 ng/ml) levels differed between the groups before start of the diets but fasting glucose levels were subsequently generally lower in HFD Tg compared to wt mice (Fig. 3a). The reduction in glucose levels in the wt HFD mice at weeks 12 and 17 is probably a consequence of the different phenotyping studies performed leading to some reduction in body weights (Fig. 1c).

To examine if the lower glucose levels in the Tg mice were due to lower hepatic glucose production, we evaluated hepatic gluconeogenesis with an intra-peritoneal Pyruvate Tolerance Test (PTT).

Hepatic glucose production was indeed lower in HFD Tg than wt mice and similar to LFD mice (Fig. 3b). This was not due to higher insulin levels inhibiting hepatic glucose production since also fasting insulin levels were lower in HFD Tg than in wt mice (Fig. 3c). Consistent with this, HFD Tg mice had an improved insulin sensitivity in both the intra-peritoneal Glucose Tolerance (GTT) and Insulin Tolerance Tests (ITT) (Fig. 3d,e) where HFD Tg mice were similar to LFD wt mice.

### Euglycemic clamps verify increased insulin sensitivity and peripheral glucose uptake in Tg mice

**Figure 4:**
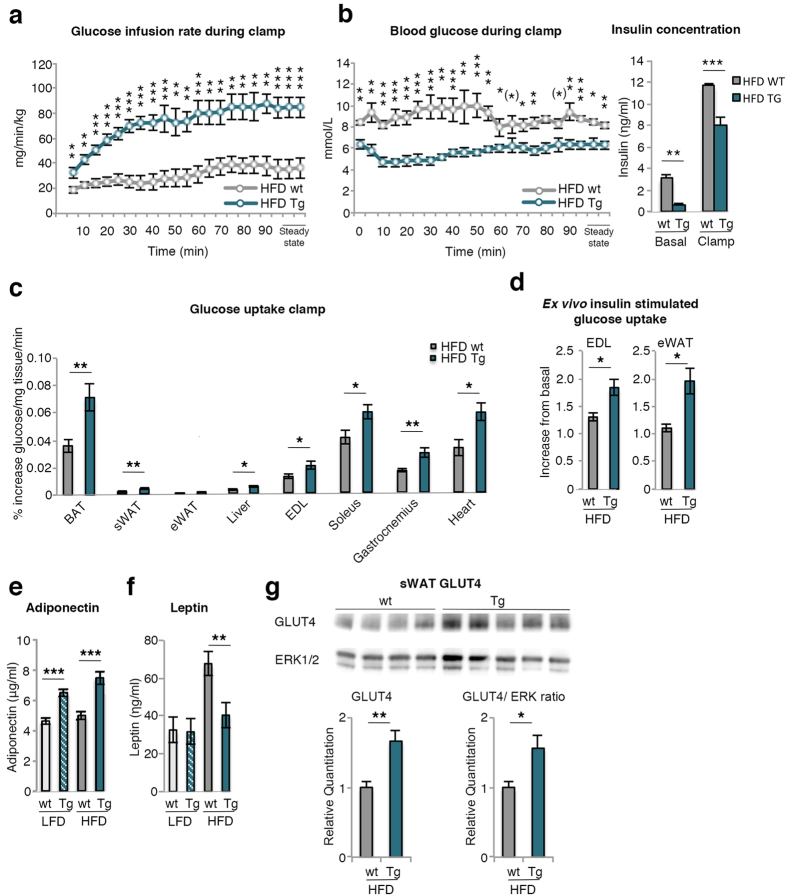
Hyperinsulinemic-euglycemic clamps, peripheral glucose uptake and serum adiponectin levels. (**a**) Glucose infusion rate, (**b**) Blood glucose and insulin concentrations during hyperinsulinemic-euglycemic clamps in conscious mice on HFD. Steady-state values are at least 5 minutes apart where the glucose did not differ more than 10%, (**c**) [C^14^]-2-deoxyglucose uptake during clamp in BAT, sWAT, eWAT, liver, extensor digitorum longus muscle (EDL), soleus, gastrocnemius and heart (n = 6–10/group). (**d**) *Ex vivo* insulin-stimulated glucose uptake in EDL and eWAT measured in mice after 17 weeks on HFD (n = 3–6/group). (**e**) Serum levels of adiponectin and (**f**) Leptin were measured in mice after 17 weeks (n = 7–18/group). (**g**) GLUT4 protein in isolated mature sWAT from wt and Tg HFD mice (top) and related to ERK as loading control (below) (n = 4–5/group). Full-length blots are presented in Supplementary Fig. 7c. The experimental data are presented as means ± SEM. 2-way ANOVA was used to compare ≤4 groups; otherwise Student’s t-test was used. ***p < 0.001, **p < 0.01, *p < 0.05, (*)p < 0.1.

To further validate an increased whole-body insulin sensitivity, HFD wt and Tg mice underwent conscious euglycemic-hyperinsulinemic clamps with [C^14^]-2-deoxyglucose tracer. These results confirmed a markedly improved peripheral insulin sensitivity in HFD Tg mice. In spite of the significantly higher insulin levels during the clamp in the wt mice, glucose infusion rates were markedly lower and, yet, the blood glucose levels were somewhat higher during the clamp (Fig. 4a,b). Taken together, these results clearly demonstrate the increased peripheral insulin sensitivity in the HFD Tg mice including both the liver and skeletal muscles.

To further examine tissues responsible for the increased insulin-stimulated glucose uptake during the clamp, we measured [C^14^]-2-deoxyglucose uptake in heart, muscles and BAT/WAT. All these tissues had significantly increased insulin-stimulated glucose uptake in HFD Tg mice (Fig. 4c) and this increase was also confirmed *ex vivo*. Insulin-stimulated glucose uptake in both isolated extensor digitorum longus (EDL) muscle and epididymal adipocytes were higher in Tg cells (Fig. 4d) showing that the increased glucose uptake is not secondary to the ambient milieu but is a cell-autonomous effect.

### Increased GLUT4, markers of adipose cell lipogenesis and adiponectin in Tg mice

Transcriptional activation of genes involved in insulin-stimulated glucose uptake in adipose tissue is shown in Table 2 and in skeletal muscle and liver in Supplementary Table 2. The key insulin-regulated glucose transporting protein *Glut4* was significantly increased in sWAT, eWAT and skeletal muscle of HFD Tg mice when compared to wt mice and similar to that of lean wt or Tg mice (not shown). Thus, obese Tg mice maintained their *Glut4* expression while it was downregulated in HFD wt mice as also previously reported^20^,^21^. *Adiponectin* was also increased in both adipose tissue depots and so were the serum levels in both LFD and HFD Tg mice (Fig. 4e). Leptin serum levels were increased in HFD mice but less so in Tg mice (Fig. 4f) probably due to the smaller adipose cells since cell size is an important determinant of leptin secretion^22^.

Markers of adipose cell differentiation, including GLUT4 protein (Fig. 4g)*, Insr, Bmp4*, and lipogenic genes were increased in both adipose tissue depots whereas *Bmp2* and *Bmp7* were unaffected (Table 2). GLUT4 is an important regulator of adipose cell lipogenesis through ChREBP activation, including the novel family of secreted lipids Fatty Acid esters of Hydroxy Fatty Acids (FAHFAs), shown to be anti-inflammatory and to improve peripheral tissue insulin sensitivity^23^. Markers of beige and brown adipose cells were not changed in sWAT including the recently identified thermogenic protein SLIT2 secreted by beige adipose cells^24^. Inflammatory markers were reduced which is consistent with the smaller adipose cell size in HFD Tg while markers of TGFβ activation such as *Tgfb1* and *ctgf* were essentially unchanged and a similar profile was seen in eWAT (Table 2). We also examined other ligands for TGF activation including InhibA (Activin), shown to enhance proliferation of adipose precursor cells but inhibit their differentiation^25^, which was reduced while follistatin, an inhibitor of the TGF ligand myostatin^26^, was increased. Together these data strongly indicate that TGFβ activation does not account for the increased number of adipose cells seen in Tg mice.

We also analyzed gene expression in the liver, but there was no evidence of altered lipogenesis (Supplementary Table 2).

### Increased serum FAHFAs in HFD Tg mice

**Figure 5:**
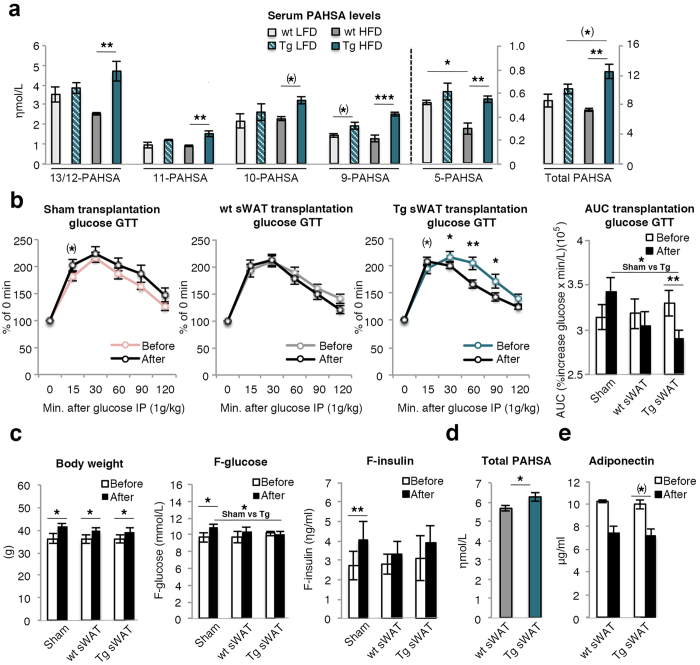
Serum levels of PAHSAs and characterization of glucose and insulin levels before and after transplantation. (**a**) Quantification of PAHSA isomers in serum. The number of the PAHSA refers to the location of the ester bond in the PAHSA isomer (n = 3/group). (**b**) Glucose values, (**c**) body weight, fasting glucose and fasting insulin values from intraperitoneal glucose tolerance test (GTT) before (6 weeks on HFD diet) and 2 weeks after transplantation at week 8 (i.e.; 10 weeks on HFD diet) of 0.8 g subcutaneous adipose tissue (sWAT) from wt/Tg mice placed in the abdominal cavity. GTT is calculated as percentage of the fasting value at time point 0. AUC was calculated from the GTT curve (n = 6–8/group). (**d**) Quantification of total serum PAHSA isomers in recipient mice transplanted with wt or Tg sWAT for 2 weeks (n = 7–8/group). (**e**) Serum levels of adiponectin before and after transplantation (n = 7–8/group). The experimental data are presented as means ± SEM. 2-way ANOVA was used to compare ≤4 groups; otherwise Student’s t-test was used. ***p < 0.001, **p < 0.01, *p < 0.05, (*)p < 0.1.

Since GLUT4 and carbohydrate-responsive-element-binding proteins (*Chrebp*; also known as *Mlxipl*) and other markers of lipogenesis were increased in the adipose tissues of Tg mice, we measured serum levels of FAHFA isomers palmitic acid-hydroxy stearic acids (PAHSA) 13/12-, 11-, 10-, 9- and 5- PAHSA in wt and Tg mice. We have previously shown that this novel family of lipids are circulating in the blood, have positive effects on insulin sensitivity and are reduced in obesity^23^. We also found serum levels of 13/12- and 5- PAHSA to be reduced in obese wt mice but, in contrast, increased in the HFD Tg mice and similar to those in LFD wt mice (Fig. 5a). Thus, the increased insulin sensitivity in HFD Tg mice can be a consequence of the hypercellular adipose tissue with increased GLUT4, lipogenesis and increased secretion of FAHFAs.

### Transplanting Tg subcutaneous fat improves glucose tolerance and insulin sensitivity in obese recipient mice

We then wanted to validate if Tg subcutaneous adipose tissue secreted factors such as adiponectin or FAHFAs which, in an endocrine fashion, improved insulin sensitivity in obese recipient mice. Transplantation of 0.8 g subcutaneous adipose tissue from Tg mice to recipient mice improved their glucose tolerance when measured after 2 weeks compared to sham-operated mice where the glucose tolerance tended to be lower probably due to their increased body weights. Furthermore, Tg subcutaneous adipose tissue was significantly better in improving the glucose tolerance in recipient mice than wt tissue (Fig. 5b,c). This effect was seen in spite of similar insulin levels supporting an improved insulin sensitivity in spite of the quite small amount of adipose tissue that was transplanted.

We also measured if the serum PAHSA or adiponectin levels were increased in the recipient mice of wt and Tg adipose tissue, thereby supporting that these molecules were secreted by the adipose tissue and improved insulin sensitivity. Although the individual PAHSA levels were not significantly different, total PAHSA levels were significantly increased in mice transplanted with Tg compared to wt mice adipose tissue (Fig. 5d). This effect was small and does not by itself prove causality but is consistent with the positive effects on insulin sensitivity following administration of PAHSAs to mice *in vivo*^23^. In contrast, adiponectin levels were actually reduced in both groups of the transplanted mice probably due to their expanded adipose tissue mass (Fig. 5e).

We also transplanted brown adipose tissue (0.1 g) from both wt and Tg mice. However, there was no difference between the wt and Tg transplants in improving the glucose tolerance in the recipient mice (Supplementary Fig. 4a). Importantly, there was no difference in area under the curve among the different groups before the transplantation (Supplementary Fig. 4b).

Taken together, these results support a role of endocrine factors secreted by the adipose tissue in improving insulin sensitivity in HFD Tg mice and where PAHSAs rather than adiponectin are strong candidates.

### WISP2 Tg mice serum increases mesenchymal precursor cell proliferation

**Figure 6:**
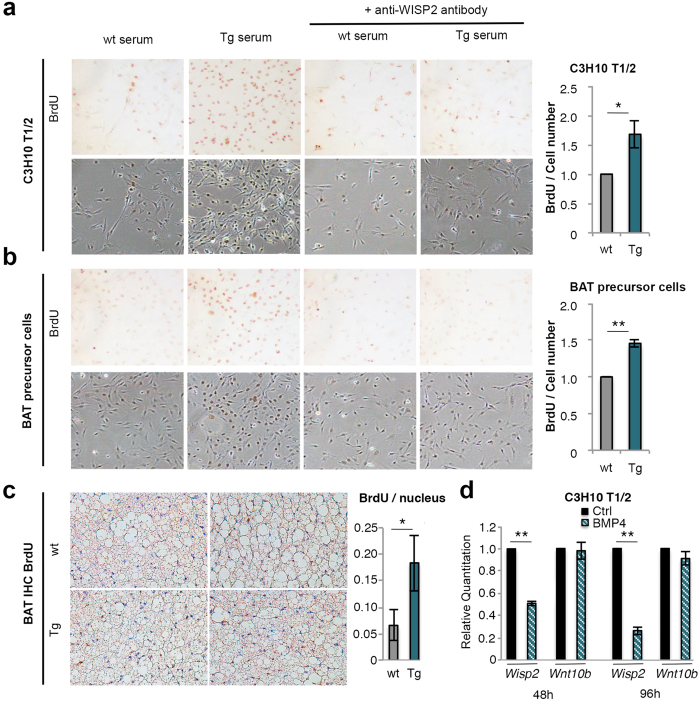
Proliferation of mesenchymal precursor cells and inhibition of *Wisp2* by BMP4. (**a**) C3H10 T1/2 cells and (**b**) BAT precursor cells were incubated with serum from mice fed HFD for 17 weeks and pre-incubated with or without monoclonal WISP2 antibodies. Proliferation is shown by BrdU incorporation (upper panels), light microscopy pictures (lower panels) (10 x magnification) and quantified (n = 3/group). (**c**) Adjacent BAT sections of aged adult mice were visualized by BrdU incorporation and Hematoxylin staining and quantified (n = 3/group). (**d**) Expression of *Wisp2* and *Wnt10b* in C3H10 T1/2 cells treated with or without 50 ηg/ml BMP4 (n = 4/group). The experimental data are presented as means ± SEM. Student’s t-test was used. **p < 0.01, *p < 0.05.

We then wanted to validate that the increased muscle, heart and adipose tissue mass and hyperplasia were due to WISP2 increasing the growth of precursor cells. We have previously found WISP2 to increase growth of undifferentiated murine and human preadipocytes by activating canonical WNT and MAPK^16^. Thus, we postulated that the increased secretion of WISP2 by the adipose tissue could, in an endocrine/paracrine fashion, enhance the growth of the mesenchymal precursor cells in the tissues but with maintained normal signals for termination of growth and induction of cell differentiation. To examine if WISP2 in the serum alters mesenchymal precursor cell proliferation, we incubated the murine multipotent C3H10 T1/2 mesenchymal stem cells with serum from either wt or Tg animals. Tg serum clearly increased cell proliferation (Fig. 6a). To verify that this was due to WISP2 protein, we pre-incubated Tg serum with monoclonal anti-WISP2 antibodies before addition to the cell culture. This pre-incubation almost completely prevented the proliferative effect by Tg serum (Fig. 6a).

Since a marked hyperplastic effect also was seen in BAT, we repeated the same experiment with BAT precursor cells. The proliferative effect of Tg serum was even more pronounced and again inhibited by the monoclonal anti-WISP2 antibodies (Fig. 6b). We also examined the effect in human preadipocytes and both rhWISP2 and transfecting the cells with full-length WISP2 increased their proliferation similar to the murine cells (Supplementary Fig. 5).

We also verified that HFD Tg mice had increased growth of BAT cells *in vivo* by injecting mature mice with BromodeoxyUridine (BrdU). BAT from Tg mice was hypercellular, had smaller lipid droplets than in wt as well as increased BrdU incorporation documenting increased proliferation also *in vivo* (Fig. 6c).

Taken together, these results strongly support the concept that WISP2 as a secreted protein by the adipose tissue in Tg mice enhances the growth of mesenchymal precursor cells and tissue expansion. This is consistent with the expanded, pro-mitotic and hypercellular WAT, BAT and heart and it probably also applies to the skeletal muscles although not specifically examined. This is also consistent with the particular expansion of “visceral” epididymal and mesenteric fat in Tg mice since these depots can expand at a later stage while cellular expansion of the subcutaneous adipose tissue is restricted to the first 6 weeks of life^18^,^19^.

### WISP2 is not an inhibitor of TGFβ in adipose precursor cells

TGFβ is increased in obesity^27^ and TGF ligands such as Activin have been shown to increase preadipocyte proliferation and inhibit their differentiation^25^ but, as shown in Table 1, there was no evidence of altered TGF signaling in WAT of Tg mice. However, this differs from previous results in cancer cells and heart fibroblasts where WISP2 is a powerful inhibitor of TGFβ activation, inhibiting epithelial-mesenchymal cell transition^11^ as well as the development of myofibroblast-induced fibrosis when expressed in the heart^12^. WISP2 was also found to be reduced in hearts from patients with fibrotic heart failure^12^. To further examine this, we overexpressed WISP2 or incubated the mesenchymal stem cell-like NIH C3HT101/2 cells with WISP2 protein and/or WISP2 siRNA with TGFβ for different times. As shown in Supplementary Fig. 6a-c pSMAD2 activation was not altered by any of these procedures supporting the concept that WISP2 does not promote growth of mesenchymal precursor cells through TGFβ activation^16^.

### WISP2, but not WNT10b, is inhibited by BMP4 in adipose precursor cells

As a canonical WNT activator, we expected a different phenotype in the WISP2 Tg mice than that seen. A previous mouse model expressing the canonical WNT ligand WNT10b in the adipose tissue under an aP2 promoter^28^ showed a completely different phenotype than our WISP2 model. Those mice had *reduced* amounts of WAT and completely lacked BAT. Since WISP2 is a non-conventional WNT activator, we explored possible mechanisms for the apparently normal endogenous precursor cell commitment and regulation of adipogenesis in WISP2 Tg mice. Our results are consistent with endocrine effects of adipose-tissue secreted WISP2 enhancing the growth of mesenchymal precursor cells and, thus, primarily mediated through extracellular signals. A major regulator of mesenchymal precursor cell adipogenic commitment is BMP4^15^ and BMP4 is secreted by differentiated adipose cells targeting tissue precursor cells in a paracrine fashion^29^. BMP4 was upregulated in Tg adipose tissue and we, therefore, compared the effect of BMP4 on transcriptional regulation of *Wisp2* and the conventional canonical WNT ligand *Wnt10b* used in the transgenic mice^28^.

We tested the effect of BMP4 in both CH3T10 1/2 cells and human undifferentiated preadipocytes. As shown in Fig. 6d, BMP4 rapidly inhibited *Wisp2*, but not *Wnt10b*, mRNA levels and similar results were obtained with human preadipocytes (data not shown). Thus, we conclude that WISP2 is a secreted growth-promoting protein for undifferentiated mesenchymal precursor cells but it is subject to the normal regulation of adipogenic commitment by BMP4 while conventional WNT ligands, like WNT10b, are not inhibited. This is an important difference, which can explain the normal adipose tissue growth in WISP2 but not in WNT10b Tg mice.

## Discussion

WISP2 Tg mice showed a completely different phenotype than the lipodystrophic Tg mouse model overexpressing the conventional canonical WNT ligand WNT10b in the adipose tissue^28^ or an activated β-catenin in adipose precursor cells^30^. WISP2 Tg mice were characterized by; increased lean body mass, increased whole-body energy expenditure as a consequence of increased lean body mass, improved insulin sensitivity, increased proliferation and growth of mesenchymal precursor cells leading to markedly increased and hyperplastic BAT, “healthy” hyperplastic WAT and larger hyperplastic hearts. This phenotype in the Tg mice was maintained for the entire observation period and Supplementary Fig. 2a shows that also chow-fed Tg mice maintained the increased growth over 52 weeks.

These findings show that WISP2 is an adipose tissue endogenous and secreted growth factor enhancing proliferation of mesenchymal precursor cells, documented both *in vivo* and *ex vivo.* The transgene targeted the adipose tissue and the local concentrations in the adipose tissue and differentiated adipose cells were increased (Fig. 1a,b). The increased heart and skeletal muscle mass in Tg mice were most likely due to endocrine effects of secreted WISP2 since heart and muscle WISP2 protein concentrations were not increased. However, WISP2 does not seem to target and/or reach sufficiently high local levels around bone precursor cells since there was no difference in bone density or growth. If this is different in older mice where bone marrow adipose cells are increased and where local WISP2 secretion may be higher requires further studies.

Interestingly, WISP2 is not only highly expressed in adipose tissue precursor cells but also high in C2C12 muscle cells, human myoblasts as well as in osteoblasts (data not shown). The role and regulation of WISP2 in these precursor cells is currently under examination.

There are several differences between WISP2 and other established and conventional canonical WNT ligands such as WNT10B and WNT3A. WISP2 activates canonical WNT signaling and phosphorylates the LRP5/6 co-receptor but, in contrast to conventional WNT ligands, it does not need to be acylated/palmitoylated to be secreted and, thus, should not be able to bind to the Frizzled receptors^16^. This selectivity in action has also previously been found for other ligands including TGFβ, PDGFα and another member of the CCN family with a high degree of homology to WISP2; CTGF^31^.

WISP2, in contrast to the other WNT ligands, has close cross-talk with BMP4 which directly inhibits the transcriptional activation of WISP2 and allows the cells to enter adipogenic differentiation. BMP4 is also highly expressed and secreted by differentiated adipose cells targeting tissue precursor cells to induce their commitment/differentiation as adipose cell size increases^29^.

WISP2 has been shown to inhibit TGFβ and epithelial- mesenchymal cell transformation (EMC) in certain cancer cells and to inhibit TGFβ-induced myofibroblast formation and fibrosis in the heart^12^. However, WISP2 is a multidomain and multifunctional protein with cell-specific effects. We examined if WISP2 inhibits TGFβ in the multipotential stem-cell like NIH C3HT101/2 cells but saw no such effect. Similarly, we saw decreased levels of the TGF ligand Activin/InhibinA which increases adipose precursor cell proliferation but inhibits their differentiation^25^. Thus, we conclude that WISP2 enhances mesenchymal precursor cell growth as a BMP4-regulated canonical WNT-associated growth factor.

In spite of similar or even increased amounts of some WAT depots, the “healthy” hyperplastic adipose tissue profile in Tg mice was associated with considerably higher serum and adipose tissue mRNA levels of adiponectin. Serum levels of leptin were increased in HFD wt animals but essentially unchanged in HFD Tg mice probably due to the hypercellular rather than hypertrophic adipose tissue. Adipose cell size is an important regulator of leptin secretion^22^. Markers of lipogenesis, *Glut4* and *Chrebpa/b* were increased in the adipose tissue and glucose uptake *ex vivo* was also increased in Tg adipose cells showing that this was a cell-autonomous effect and not dependent on the milieu. WISP2 itself does not increase GLUT4 or ChREBP in cultured (pre)adipocytes (data not shown). Thus, it is more likely that the increased GLUT4, *Chrebp* and lipogenic markers are secondary to the increased hyperplastic proliferation and differentiation of the adipose cells leading to an adipose tissue with smaller and well functional “healthy” cells. Increased lipogenic markers in the adipose tissue, together with improved insulin sensitivity, were also seen in other obese mouse models of “healthy” hyperplastic adipose tissue, i.e., Tg mice overexpressing adiponectin^5^ or GLUT4^6^ in the adipose cells in spite of the fact that the animals were more obese. A similar conclusion can be inferred from another recent study comparing different genetic mouse models^32^. Thus, enhancing hyperplasia in the expanding adipose tissue overcomes the negative consequences of increased body fat with dysfunctional hypertrophic adipose cells.

Upregulation of GLUT4 in the adipose tissue is associated with increased lipogenesis and biosynthesis of lipids regulated by CHREBPβ. This includes the secretion of a novel family of fatty acid esters of hydroxy fatty acids (FAHFAs) with significant positive effects on both inflammation and insulin action^23^. We here also found that both *Chrebpa* and *Chrebpb* were upregulated in the adipose tissue in WISP2 Tg mice and, consistent with this, we found that obesity induced by HFD was associated with lower 13/12- and 5-PAHSA as previously described^23^ while the levels in the HFD Tg mice were at least as high as in non-obese mice.

We also transplanted subcutaneous adipose tissue to obese recipient mice and found that this improved the glucose tolerance as also previously reported when transplanting adipose tissue from control and exercised mice^33^. However, Tg adipose tissue was markedly better than wt tissue and the serum FAHFA levels were also increased in the recipient mice receiving Tg fat although this was a small difference, probably due to the small amount of adipose tissue transplanted (0.8 g). However, the serum levels of adiponectin, another adipokine with positive effects on insulin sensitivity, were actually reduced probably as a consequence of the increased body weights following the transplantations. Together, our results support the concept of endocrine factors released by the adipose tissue improved insulin sensitivity and strengthen the possibility that the FAHFAs play a role in mediating the improved insulin sensitivity seen in both the Tg mice and the recipient mice following transplantation.

In conclusion, WISP2 is a novel secreted adipokine, most highly expressed in the subcutaneous adipose tissue and primarily in mesenchymal precursor cells. WISP2 is, to the best of our knowledge, the first identified endogenous and secreted autocrine/endocrine regulator of adipose tissue and heart/muscle cellularity and growth. The positive effects of secreted WISP2 on mesenchymal tissue hyperplasia and growth, increased circulating levels of adiponectin and PAHSAs and improved insulin sensitivity make WISP2 a novel and attractive target to prevent obesity-related metabolic complications including insulin resistance and Type 2 diabetes.

## Methods

### Generation of transgenic animals

The aP2- Wisp2 Tg mice were created in the laboratory of Fatima Bosch (Center of Animal Biotechnology and Gene Therapy, Universitat Autònoma de Barcelona, Bellaterra, Spain) using microinjection of oocytes from C57Bl6/SJL mice. Tg founders were then bred to generate F1 Tg mice and potential founders were screened for transgene integration by Southern blot and PCR analysis from tail DNA. The Tg F2 offspring was generated by backcrossing the F1 Tg mice with wt C57BL/6NTac mice (Taconic, Lille Skensved, Denmark) for 10 generations and then inbred for 4–7 generations (B6N.SJL/J-Tg(aP2-Wisp2)92Fbos; N10,F4-F7).

### Animals

Only male mice were used for phenotyping. Animals were weaned at 3 weeks of age and housed 2–5/cage in a temperature-controlled (21 °C) facility with a 12-h light-dark cycle with free access to chow food and water. From the age of 6 weeks, age-matched male Tg mice and wt littermates were fed either pelleted HFD (45 kcal% fat; D12451 or D12451i; Research Diets, New Brunswick, NJ, USA), pelleted LFD (10 kcal% fat; D12450B; Research Diets) or kept on chow diet (CD) for up to 52 weeks.

Total body weight was recorded weekly during the period of 17 weeks on LFD or HFD or every 8th week on CD. CD animals were injected daily intraperitoneally with 50 mg/kg BrdU (Sigma-Aldrich, Saint Louis, MO, USA) for 5 days before termination.

At the end of the study period, mice were euthanized using 5% isofluorane (Baxter Medical AB, Kista, Sweden) and blood was collected by heart puncture for analysis of metabolites. Tissues were dissected, weighed and snap-frozen in liquid nitrogen or treated appropriately for further analysis.

All animal experiments were performed in accordance with guidelines and regulations from the Gothenburg Animal Ethics Committee. Experimental protocols were approved at the Administrative Court of Appeals in Gothenburg, Sweden.

### Blood chemistry

Fasting (4 hours food withdrawal) glucose and insulin concentrations were measured in blood samples taken from the tip of the tail or the submandibular vein^34^. Glucose and insulin were measured using an Accu-Check glucometer (Roche Diagnostics, Basel, Switzerland) and Ultrasensitive Mouse Insulin ELISA kit (Chrystal Chem Inc., Downers Grove, IL, USA), respectively.

Plasma adiponectin was quantified by a mouse Adiponectin/Acrp30 Quantikine ELISA Kit (R&D Systems, Inc., Minneapolis, MN, USA) and plasma leptin by Mouse Leptin ELISA Kit (Chrystal Chem Inc.). Serum triglycerides and cholesterol were measured in whole plasma using an Infinity Triglyceride and Infinity Cholesterol kit (Thermo Fischer Scientific, Waltham, MA, USA).

### Body composition

Analysis of total body fat and lean body mass was performed by dual energy X-ray absorptiometry (DEXA) using the Lunar PIXImus II Densitometer with version 2.10.041 software (GE Healthcare Life Sciences, 259 Waukesha, WI, USA)^35^. CT scans were performed with the pQCT XCT RESEARCH M (version 4.5B, Norland Medical Systems Inc., Fort Atkinson, WI, USA) operating at a resolution of 70 μm, as described previously^36^.

### Indirect calorimetry

Respiratory exchange quotients (RQ) were estimated using the indirect calorimetry system INCA (Somedic AB, Hörby, Sweden). Oxygen consumption (VO2) and carbon dioxide production (VCO2) were recorded every 2 min for 23 h. Animals had ad libitum access to food and water during the measurements. The data for the first hour were discarded to account for animal acclimatization to the testing conditions. RQ was calculated per hour as the VCO2/VO2 ratio.

### Open field activity test

This test was performed to study locomotor activity and food intake as described previously^37^ (Kungsbacka Mät and Reglerteknik AB, Fjärås, Sweden). The amount of food was measured before and after the test and daily food intake per animal calculated.

### Glucose tolerance tests (GTTs), insulin tolerance tests (ITTs) and pyruvate tolerance tests (PTTs)

Following 4 h of food withdrawal, mice were injected with glucose (1 g/kg; Fresenius Kabi, Bad Homburg, Germany), human recombinant insulin (0.8 U/kg; Actarapid Penfill, Novo Nordisk, Bagsværd, Denmark) or sodium pyruvate (2 g/kg; Sigma-Aldrich) intraperitoneally at time 0. Blood was taken from the tail tip, saline solution (9 mg/ml; Fresenius Kabi), insulin (0.8 U/kg) or glucose (1 g/kg), was given as fluid replacement or to compensate for high/low plasma glucose.

### Euglycemic-hyperinsulinemic clamp studies in conscious mice

Surgery and glucose and insulin infusion were performed as previously described^37^ using an insulin infusion rate of 7.5 mU/min/kg. When the steady state was reached (t = ~90 min), a bolus of 2[^14^C]deoxyglucose (2DOG) (3 μCi; PerkinElmer, Waltham, MA) was injected through the jugular vein. Blood was sampled at 93, 96, 100, 105, 110, 120, 130, and 150 min postinjection. Mice were then killed and tissues analyzed for glucose uptake as previously described^38^. The accumulation of 2[^14^C]deoxyglucose-6-phosphate in the tissue was normalized to the tissue weight and normalized to the area under the plasma [^3^H]-2DOG decay curve divided by blood glucose concentration.

### Transplantation of sWAT and BAT

Transplantation was performed as previously described^33^. 6 weeks old C57BL/6N mice (Taconic) were put on a HFD diet (60 kcal% fat; D12492; Research Diets) for 10 weeks. At week 6 and 10, GTT was performed and at week 8 sWAT (0.8 g) or BAT (0.1 g) was transplanted from 18 months old donor wt or Tg mice into the abdominal cavity.

### Isolation of adipocytes

Isolation was performed essentially as previously described^9^,^37^. Briefly, biopsies were washed to remove traces of blood and treated with collagenase (1 mg/ml) (Sigma-Aldrich) for 45–60 min at 37 °C in a shaking water bath. Isolated adipocytes were filtered through a 250 μm nylon mesh and washed with fresh medium. Cell size was measured and the remaining isolated adipocytes where either used for protein extraction or glucose uptake experiments.

### Glucose uptake

Isolated adipocytes from eWAT, sWAT and intact EDL (n ≥ 3/group) were transferred to vials with glucose-free Hank’s medium 199 (Invitrogen Corporation, Paisley, UK) supplemented with 4% BSA (Sigma-Aldrich), 0.15 μM adenosine (Sigma-Aldrich), pH 7.4. Insulin (10 ηM for adipocytes, 100 ηM for EDL; Actarapid Penfill, Novo Nordisk) was added for 15 min followed by D-[U-^14^C]-glucose (0.26 mCi/l, 0.86 μM; Amersham Biosciences GE Healthcare, Buckinghamshire, UK) for 45 min. Isolated adipocytes were separated from the medium through centrifugation and transferred to scintillation tubes to measure incorporated radioactivity. EDL muscle was immediately after incubation washed with cold PBS 3 times and digested in 0.5 M NaOH at 50 °C for 30 min. Cleared protein lysates were used to measure incorporated radioactivity by liquid scintillation counting (Beckman 6500, Brea, CA, USA).

### Histological analysis and immunohistochemistry

Liver, BAT and heart were immediately after dissection fixed in 4% phosphate-buffered formaldehyde (Histolab Products AB, Gothenburg, Sweden) and embedded in paraffin. Six mm sections were stained with hematoxylin and eosin.or incubated with dystrophin antibody (ab15277, Abcam, Cambridge, UK). BAT sections were incubated with UCP-1 antibody (ab10983, Abcam), followed by a secondary biotinylated antibody and diaminobenzidine staining (Vector Laboratories Inc., Burlingame, CA, USA) and counterstained with hematoxylin solution. For BrdU incorporation in BAT *in vivo*, tissue sections were then stained with BrdU antibody (Life Technologies, Carlsbad, CA, USA).

### WISP2 in serum

For the detection of secreted WISP2, mouse serum was first pretreated with agarose A/G plus beads (Santa Cruz Biotechnology, Heidelberg, Germany), then immunoprecipitated with the monoclonal mouse anti-WISP2 antibody^39^ and analyzed by Western blot.

### Cell proliferation assays

The BrdU proliferation assay kit (GE Healthcare) was used. C3H10 T1/2 mesenchymal cells and brown adipose precursor cells were cultured with mouse anti-WISP2 antibody-treated or non-treated serum collected from wt or Tg WISP2 mice for 48 h. Cells were fixed and stained with BrdU antibody.

### Cell culture and *Wisp2* silencing

C3H10T1/2 cells were transfected with either siRNA for silencing Wisp2 (s76052, Ambion, Thermo Fisher) or a scrambled negative control (Sigma Aldrich) using Lipofectamine RNAiMAX (Invitrogen, Thermo Scientific) in Opti-Mem and added to the medium at a final concentration of 40 nM. Recombinant human WISP2 protein (ab50040, Abcam) was added for 1 h prior to addition of recombinant mouse TGFβ (Novus biochemical, Littleton, CO, USA) and incubated for 30 min. Proteins were extracted and analyzed as described.

### Serum PAHSA Analysis

Concentrations of PAHSAs were measured in serum from Tg and wt mice on HFD and LFD. Lipid extraction and LC MS/MS analysis was performed as previously described^23^.

### Real Time RT-PCR

Quantitative real-time PCR was done using the ABI Prism 7900HT Sequence Detection System (Applied Biosystems) as previously described^37^. Analyses were performed in duplicates and normalized to the expression levels of Mouse Universal Reference Total RNA (Clontech Laboratories, Inc., Mountain View, CA, USA). Gene-specific primers and probes were designed using the Primer Express software or purchased on-demand (Applied Biosystems). Sequences and assay IDs are available upon request.

### Western blots and antibodies

Proteins were analyzed following electrophoresis through SDS-polyacrylamide gels (Life Technologies), immunoblotted and quantified essentially as described^9^,^16^. The following antibodies were used to quantify protein levels in serum and/or tissues: WISP2^39^, UCP-1 (MAB6158, R&D Systems), Akt (#9272, Cell Technology, Boston MA, USA), ERK1/2 (06-182, Upstate/Millipore, Temecula, CA, USA), rabbit polyclonal antiserum against GLUT4 (kindly provided by Dr Sam Cushman, NIH), Smad2 (#5339, Cell Technology) and Phospho-Smad2 (#3101, Ser465/467, Cell Technology).

### Statistical Analyses

The experimental data are presented as mean ± SEM. 2-way ANOVA was used to compare ≤4 groups; otherwise Student’s t-test (IBM SPSS Statistics, version 23. p < 0.05 was considered statistically significant.

## Additional Information

**How to cite this article**: Grünberg, J. R. *et al*. Overexpressing the novel autocrine/endocrine adipokine WISP2 induces hyperplasia of the heart, white and brown adipose tissues and prevents insulin resistance. *Sci. Rep.* **7**, 43515; doi: 10.1038/srep43515 (2017).

**Publisher's note:** Springer Nature remains neutral with regard to jurisdictional claims in published maps and institutional affiliations.

## Supplementary Material

Supplemental Information

## Figures and Tables

**Table 1:** Tissue weight (g) of 23 weeks old mice on LFD or HFD.

	LFD wt	LFD Tg	P-value	HFD wt	HFD Tg	P-value
Heart	0.126 ± 0.003	0.165 ± 0.009	0.104	0.156 ± 0.008	0.191 ± 0.012	0.021
TA	0.077 ± 0.002	0.082 ± 0.005	NS	0.079 ± 0.004	0.091 ± 0.004	0.047
EDL	0.019 ± 0.001	0.019 ± 0.001	NS	0.021 ± 0.002	0.022 ± 0.002	NS
Soleus	0.018 ± 0.001	0.019 ± 0.002	NS	0.021 ± 0.001	0.020 ± 0.001	NS
Gastrocnemius	0.362 ± 0.008	0.387 ± 0.013	0.030	0.400 ± 0.010	0.407 ± 0.008	NS
Total	0.602 ± 0.006	0.718 ± 0.012	0.019	0.651 ± 0.015	0.710 ± 0.017	0.036
BAT	0.167 ± 0.017	0.378 ± 0.056	0.042	0.527 ± 0.028	0.796 ± 0.078	0.002
eWAT	1.199 ± 0.102	1.618 ± 0.186	0.042	1.882 ± 0.123	2.515 ± 0.098	0.001
rWAT	0.301 ± 0.035	0.536 ± 0.049	NS	0.749 ± 0.104	1.176 ± 0.096	0.005
sWAT	0.707 ± 0.075	0.777 ± 0.096	NS	1.578 ± 0.122	1.879 ± 0.108	0.041
mWAT	0.330 ± 0.051	0.314 ± 0.038	NS	0.939 ± 0.091	0.816 ± 0.238	NS
WAT total	1.940 ± 0.342	3.563 ± 0.348	0.079	5.035 ± 0.514	6.472 ± 0.354	0.017

Abbreviations: wt, wild-type mice; Tg, transgenic mice; LFD, low-fat diet; HFD, high-fat diet; NS, non-significant.

TA, tibialis anterior; EDL, extensor digitorum longus muscle; BAT, brown adipose tissue; eWAT, epididymal white adipose tissue; rWAT, retroperitoneal WAT; sWAT, subcutaneous WAT; mWAT, mesenteric WAT.

The experimental data are presented as mean (g) ± SEM. 2-way ANOVA was used. n ≥ 11/group.

**Table 2:** mRNA expression in white and brown adipose tissue.

sWAT	HFD wt	HFD Tg	P-value	eWAT	HFD wt	HFD Tg	P-value
**Insulin signaling/action**				**Insulin signaling/action**			
*Glut4*	21.0 ± 1.86	31.5 ± 2.98	0.01	*Glut4*	6.18 ± 1.21	15.3 ± 1.57	0.001
*Insr*	2.24 ± 0.10	3.18 ± 0.19	0.001	*Insr*	0.73 ± 0.04	1.10 ± 0.07	0.001
**Fatty acid transport/lipogenesis**				**Fatty acid transport/lipogenesis**			
*Adiponectin*	253 ± 17.4	378 ± 34.3	0.007	*Adiponectin*	125 ± 25.3	208 ± 18.3	0–013
*Cebpa*	10.8 ± 0.66	13.8 ± 1.32	0.060	*Cebpa*	9.74 ± 1.68	19.1 ± 1.83	0.001
*Chrebpa*	1.80 ± 0.15	2.52 ± 0.78	0.010	*Chrebpa*	2.53 ± 0.23	3.63 ± 0.31	0.002
*Chrebpb*	0.09 ± 0.02	1.28 ± 0.48	0.030	*Chrebpb*	0.32 ± 0.11	0.56 ± 0.14	NS
*Elovl6*	1.19 ± 0.0.11	2.11 ± 0.0.38	0.027	*Elovl6*	6.23 ± 2.16	2.01 ± 0.24	0.080
*Fasn*	3.62 ± 0.47	6.75 ± 0.93	0.006	*Fasn*	5.70 ± 0.90	11.1 ± 1.08	0.002
*Pparg2*	89.4 ± 6.60	111 ± 13.2	NS	*Pparg2*	59.3 ± 10.5	106 ± 13.3	0.022
*Scd1*	28.7 ± 3.00	54.7 ± 5.84	0.001	*Scd1*	28.7 ± 5,98	63.9 ± 7,5	0.001
*Srebp1*	3.18 ± 0.18	4.97 ± 0.38	0.001	*Srebp1*	5.76 ± 0.40	7.73 ± 0.45	0.006
**BMPs**				**BMPs**			
*Bmp2*	3.22 ± 0.22	3.67 ± 0.33	NS	*Bmp2*	0.22 ± 0.03	0.24 ± 0.023	NS
*Bmp3*	0.72 ± 0.10	0.78 ± 0.1	NS	*Bmp3*	0.33 ± 0.05	0.24 ± 0.04	NS
*Bmp4*	0.16 ± 0.02	0.26 ± 0.02	0.001	*Bmp4*	0.29 ± 0.04	0.54 ± 0.04	0.001
*Bmp7*	0.55 ± 0.09	0.78 ± 0.16	NS	*Bmp7*	0.06 ± 0.04	0.03 ± 0.02	NS
**TGFβ activation**				**TGFβ activation**			
*Ctgf*	0.11 ± 0.01	0.15 ± 0.02	NS	*Ctgf*	0.036 ± 0.01	0.037 ± 0.01	NS
*Follistatin*	2.07 ± 0.24	3.11 ± 1.01	0.009	*Follistatin*	0.56 ± 0.11	0.42 ± 0.05	NS
*Inhibin beta A*	0.08 ± 0.01	0.05 ± 0.01	0.080	*Inhibin beta A*	0.17 ± 0.05	0.14 ± 0.04	NS
*Myostatin*	9.90 ± 4.1	2.86 ± 1.4	NS	*Myostatin*	1.45 ± 0.32	0.48 ± 0.10	0.010
*Tgfb1*	1.21 ± 0.28	1.23 ± 0.18	NS	*Tgfb1*	0.21 ± 0.03	0.14 ± 0.02	0.056
**Inflammation**				**Inflammation**			
*Saa1*	0.01 ± 0.01	0.04 ± 0.02	NS	*Saa1*	0.04 ± .0.01	0.02 ± 0.01	0.040
*Saa2*	0.06 ± 0.01	0.22 ± 0.10	NS	*Saa2*	0.07 ± 0.02	0.03 ± 0.01	0.050
				**BAT**	**HFD wt**	**HFD Tg**	**P-value**
**Beige/Brown marker**				**Beige/Brown marker**			
*Irisin*	0.21 ± 0.07	0.30 ± 0.09	NS	*Irisin*	0.40 ± 0.11	0.23 ± 0.05	NS
*Prdm16*	2.67 ± 0.28	3.37 ± 0.40	NS	*Prdm16*	2.33 ± 0.23	2.38 ± 0.12	NS
*Th*	UD	UD	NS	*Th*	0.05 ± 0.02	0.03 ± 0.01	NS
*Tmem26*	2.35 ± 0.55	2.70 ± 0.56	NS	*Tmem26*	0.16 ± 0.11	0.05 ± 0.01	NS
*Ucp1*	0.30 ± 0.09	1.35 ± 0.41	0.020	*Ucp1*	5381 ± 616	6465 ± 353	NS

Abbreviations: wt, wild-type mice; Tg, transgenic mice; LFD, low-fat diet; HFD, high-fat diet; NS, non-significant; UD, undetermined. Glut4, Glucose transporter type 4; Insr, Insulin receptor; Cebpa, CCAAT/enhancer-binding protein alpha; Chrebpa/b, Carbohydrate-responsive-element-binding protein alpha/beta; Elovl6, Elongation of long-chain fatty acids family member 6; Fasn, Fatty acid synthase; Pparg2; Peroxisome proliferator-activated receptor gamma 2; Scd1, Stearoyl-CoA desaturase-1; Srebp1, Sterol regulatory element-binding transcription factor 1; Bmp, Bone morphogenetic protein; Ctgf, Connective tissue growth factor; Tgfb1, Transforming growth factor beta-1 precursor; Saa1, Serum amyloid protein A; Prdm16, protein domain containing 16; Th, Tyrosine hydroxylase; Tmem26, Transmembrane Protein 26; Ucp1, Uncoupling Protein 1. The experimental data are presented as relative quantitiy of mean ± SEM. Student’s t-test was used, n ≥ 11/group.
