# Supplementary material for: Overexpressing the novel autocrine/endocrine adipokine WISP2 induces hyperplasia of the heart, white and brown adipose tissues and prevents insulin resistance
Source: Sci Rep. 2017 Feb 27;7:43515. doi: 10.1038/srep43515 (PMC5327486; doi:10.1038/srep43515)
Supplement: Supplemental Information [file srep43515-s1.pdf]

Supplemental Information

Title of manuscript:

Overexpressing the novel autocrine/endocrine adipokine WISP2 induces hyperplasia of the heart, white and brown adipose tissues and prevents insulin resistance

Authors:

John R. Grünberg, Jenny M. Hoffmann, Shahram Hedjazifar, Annika Nerstedt, Lachmi Jenndahl, Johannes Elvin, John Castellot, Lan Wei, Sofia Movérare-Skrtic, Claes Ohlsson, Louise Mannerås Holm, Fredrik Bäckhed, Ismail Syed, Fatima Bosch, Alan Saghatelian, Barbara B. Kahn, Ann Hammarstedt & Ulf Smith

Supplementary information includes:

Supplemental Information includes Supplemental Methods, seven figures and two tables and can be found with this article online.

Supplementary Methods

Isolation of murine peritoneal macrophages

Isolation was performed essentially as previously described[1]. Briefly, 5ml ice cold PBS supplemented with fetal calf serum was injected into the peritoneum of euthanized wildtype and transgenic mice. The fluid was collected and cell suspensioncollected at 1500 RPM for 8 min.

Cell culture and Wisp2 overexpression

For overexpression, a wild-type murine Wisp2 (pcDNA3.1-wisp2) or a negative control plasmid (pcDNA3.1) was transfected during subcultivation. 2.5 μg of the plasmid was mixed with Lipofectamine 3000 (Invitrogen, Thermo Scientific) in Opti-Mem and added to the cell suspensionin a final concentration of 1.28 μg/ml.

Supplementary Figures, Legends and Tables

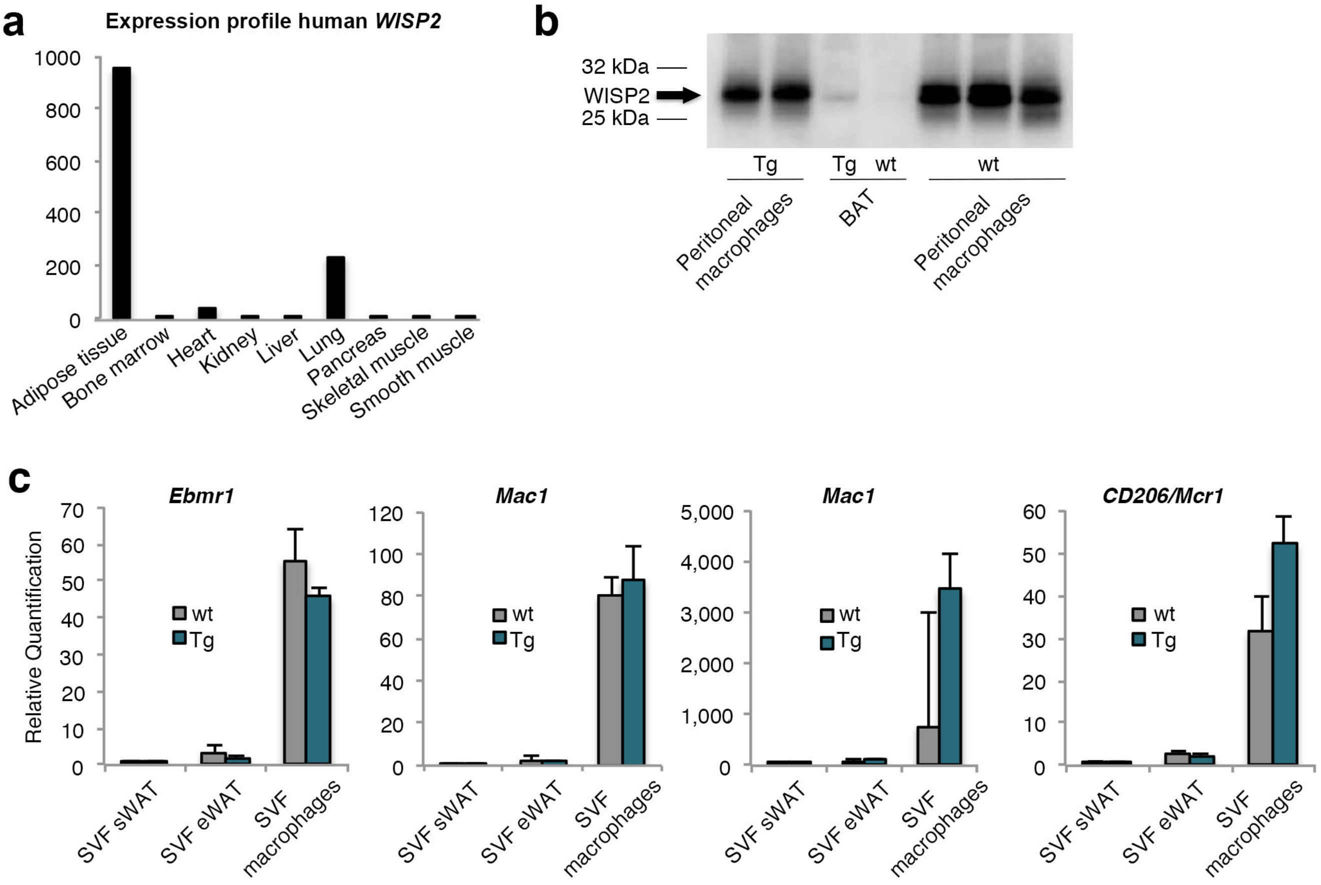

Supplementary Fig. 1. Expression profile of human WISP2 and characterization of macrophages in wildtype and transgenic mice.

(**a**)The human WISP2 gene expression (8839)[2] was retrieved from BioGPS database[3] using the HG_U133A gene atlas data set[4]. Expression values from Affymetrix chips relate to fluorescence intensity. Since there are multiple probes for each transcript on the microarray, these intensity values are summarized using various data processing algorithms, in this case gcrma. (**b**) Wisp2 protein in peritoneal macrophages of wt and Tg mice, full-length blots are presented in Supplementary Fig. 7d. (**c**) Expression of M1 and M2 genes in stromal vascular fraction(SVF) of sWAT, eWAT and macrophages (n=3-4/group). The experimental data are presented as means ± SEM. Student's t-test was used.

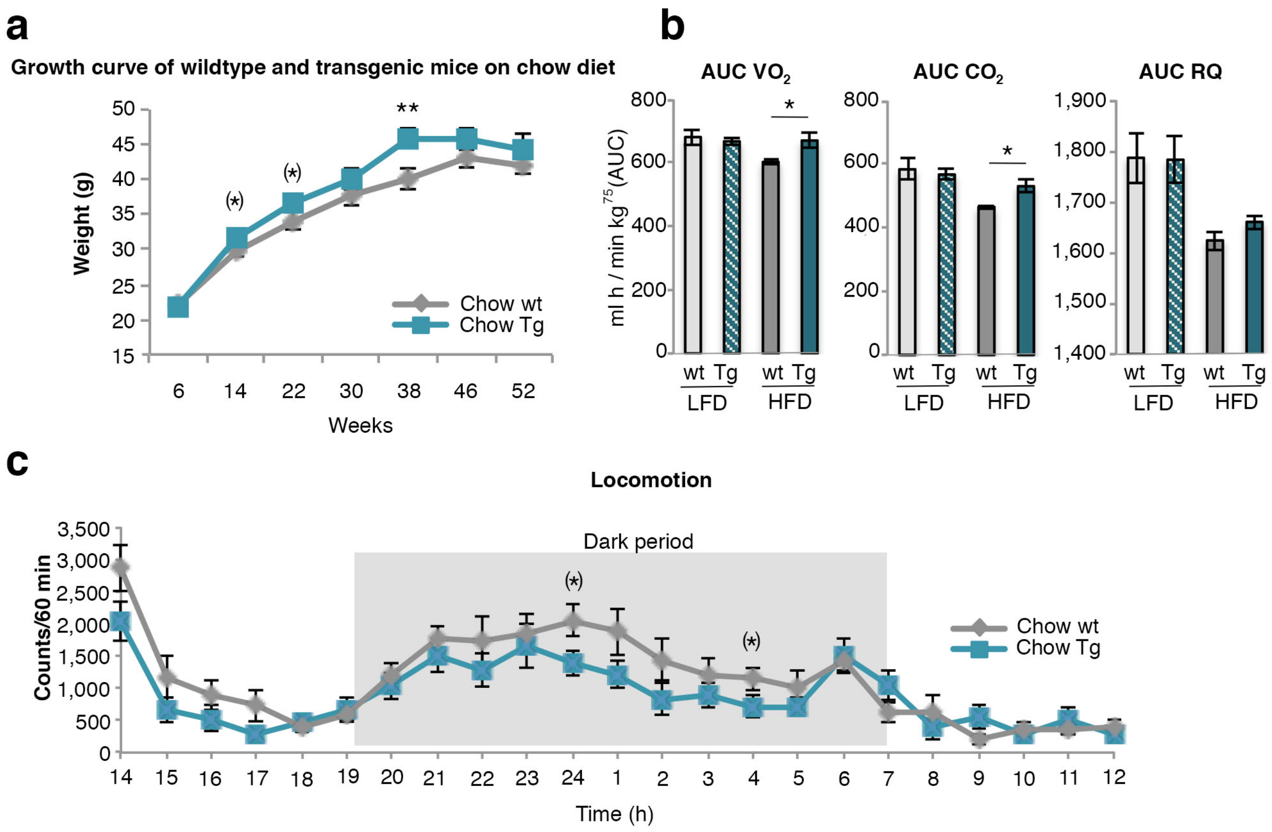

Supplementary Fig. 2. Growth curve of wildtype and transgenic mice on chow diet, indirect calorimetry per total body weight and activity patterns.

(**a**) Mice where put on chow diet for 52 weeks, weighed every 8th week **(**n=8-16/group). (**b**) Energy expenditure data normalized to total body weight are displayed as area under the curve (AUC) after 15 weeks on respective diets (n=8/group). (**c**) Activity pattern was measured as locomotion during 22h in an open field activity test on 47 weeks old mice on chow diet (n=9-11/group). The experimental data are presented as means ± SEM.2-way ANOVA was used to compare ≤4 groups; otherwise Student's t-test was used. **p<0.01, *p<0.05, (*)p<0.1.

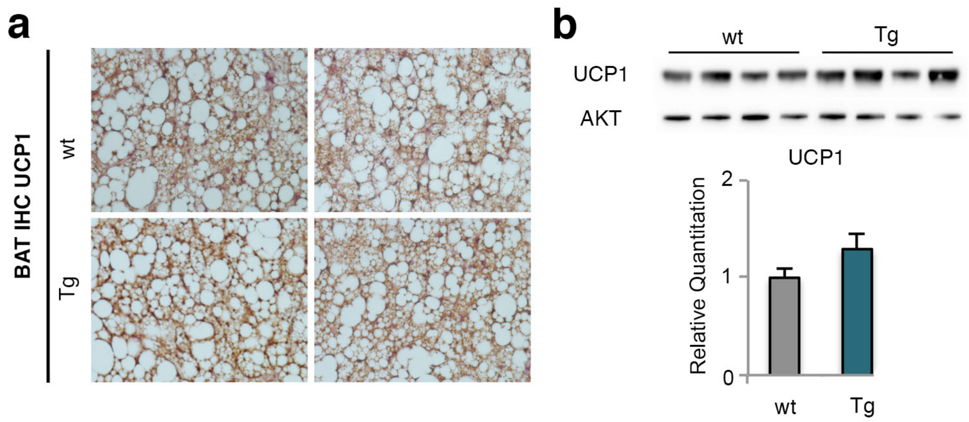

Supplementary Fig. 3. UCP1 expression in BAT.

(**a**) Adjacent BAT sections of aged adult wt and Tg mice were visualized withUCP1 antibodies and Hematoxylin staining. (**b**) UCP1 and AKT protein expression in BAT from wt and Tg mice on HFD (n=4/group). Full-length blots are presented in Supplementary Fig. 7e. The experimental data are presented as means ± SEM. Student's t-test was used.

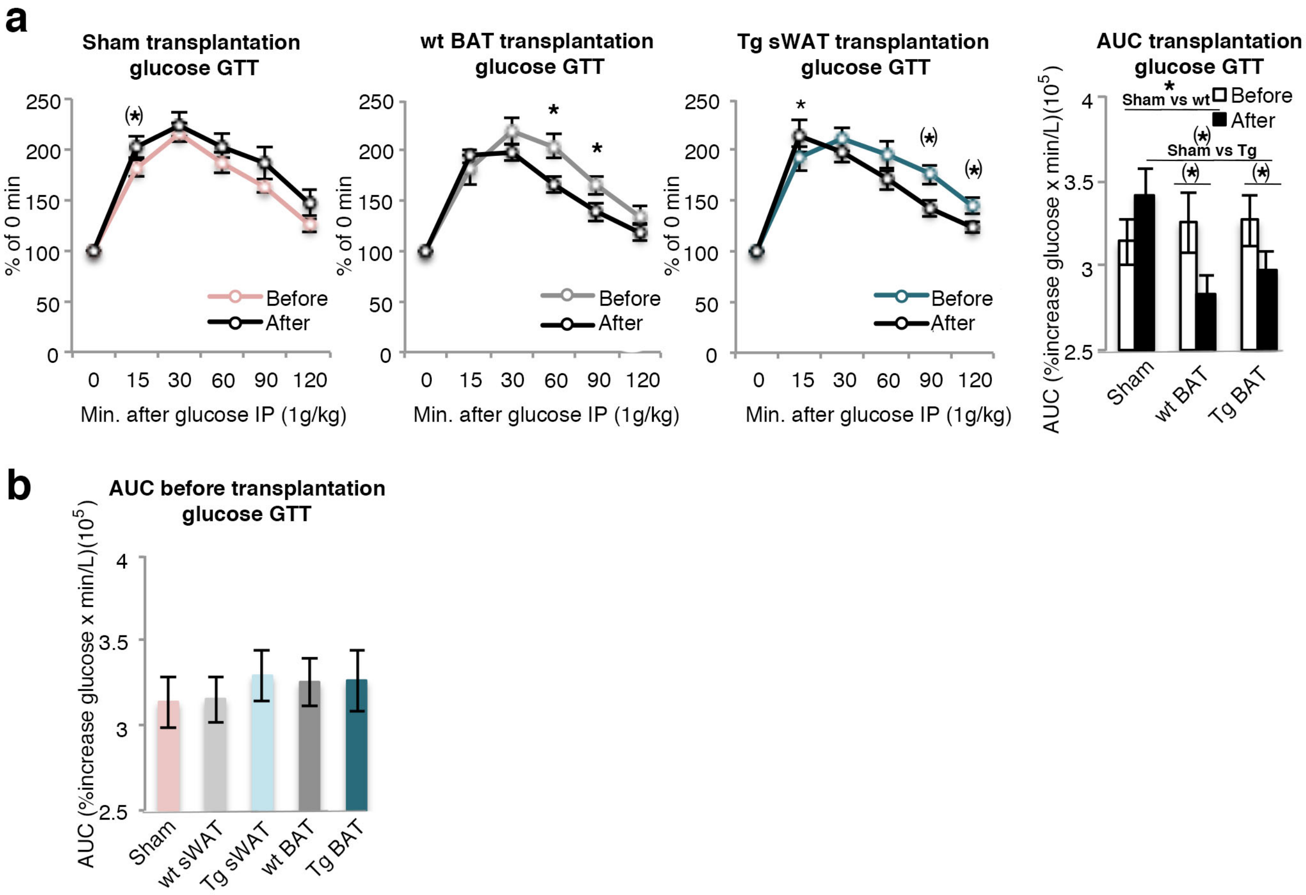

Supplementary Fig. 4. Transplantation of brown adipose tissue and AUC of glucose tolerance tests before transplantation. (**a**) Glucose values from intraperitoneal glucose tolerance tests (GTT) before (6 weeks on HFD diet) and 2 weeks after transplantation at week 8 (i.e.; 10 weeks on HFD diet) of 0.1 g brown adipose tissue (BAT) from wt /Tg mice placed in the abdominal cavity. GTT is calculated as percentage of the fasting value at time point 0. AUC was calculated from the GTT curve (n=6-8/group). (**b**) AUC of the GTTs performed before the transplantation in all 5 groups (n=6-8/group). The experimental data are presented as means ± SEM.2-way ANOVA was used to compare ≤4 groups; otherwise Student's t-test was used. *p<0.05, (*)p<0.1.

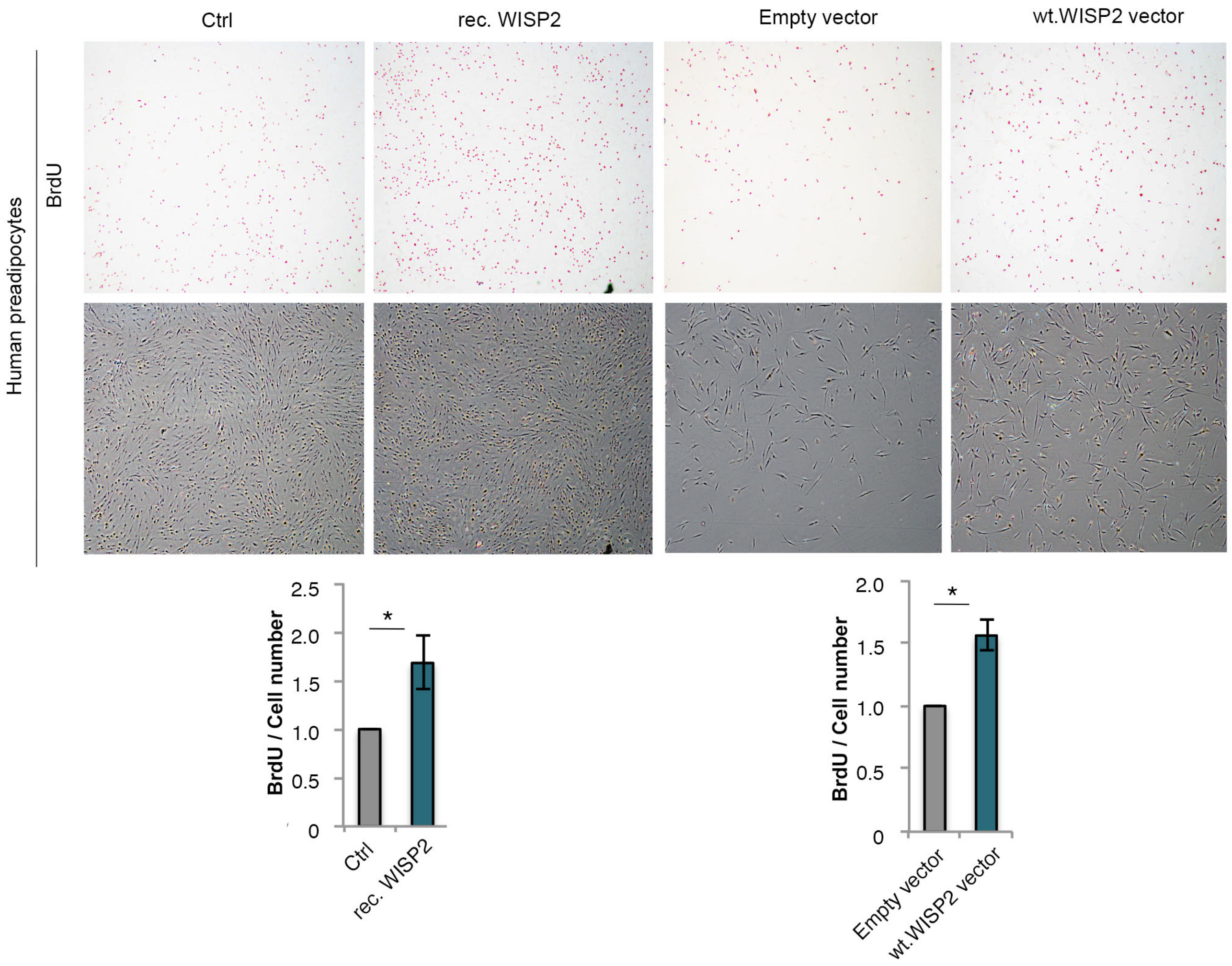

Supplementary Fig. 5. WISP2 increases proliferation of human preadipocytes.

Human subcutaneous adipose tissue preadipocytes were incubated for 72h with and without recombinant human WISP2. Preadipocytes were also transfected with empty vector or vector expressing full-length WISP2 and proliferation is shown by BrdU incorporation (upper panel) or a light microscopy picture (lower panel) (5 x magnifications) and quantified (n=3/group). The experimental data are presented as means ± SEM. Student's t-test was used. $**p<0.01$, $*p<0.05$.

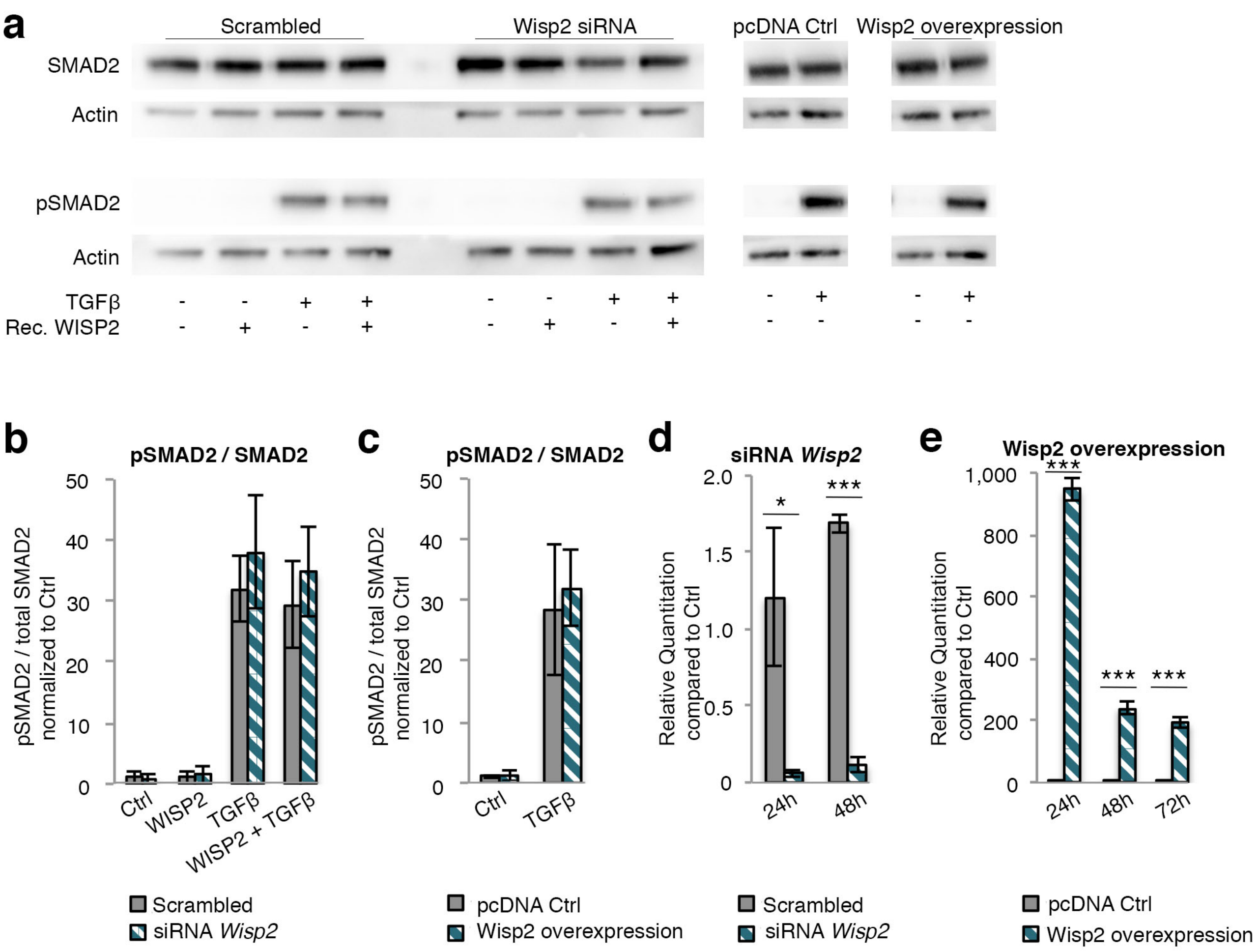

Supplementary Fig. 6. WISP2 is not an inhibitor of TGFβ SMAD signalling in mesenchymal stem cell-like NIH C3HT101/2 cells. NIH C3HT101/2 cells were incubated either with siRNA for silencing Wisp2 or a scrambled negative control. Cells were also transfected with either a plasmid overexpressing Wisp2 or a pcDNA3.1 control. Recombinant human WISP2 protein was added for 1h prior to addition of recombinant mouse TGFβ and cells were incubated for 30 min (n=3-4). (**a**) SMAD2 and Phospho-Smad2 protein,(**b-c**) quantification of the Phospho-Smad2/SMAD2 ratio. Full-length blots are presented in Supplementary Fig. 7f. Gene expression levels of *Wisp2* knockdown compared to control (**d**), or overexpression of Wisp2 (**e**), (n=4). The experimental data are presented as means ± SEM. Student's t-test was used. ***$p<0.001$, *$p<0.05$.

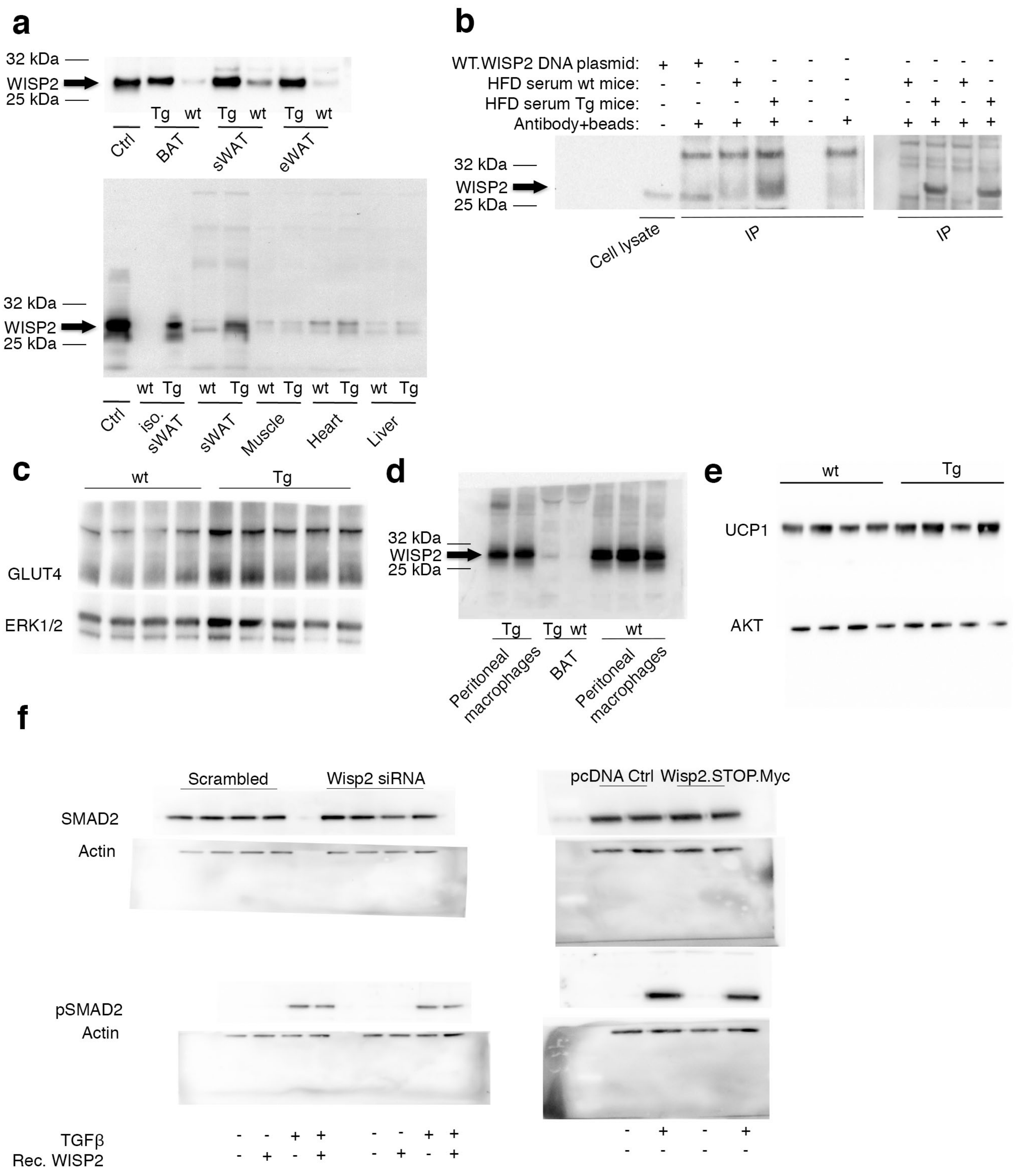

Supplementary Fig. 7. Full size Western blots. (**a**) The full-length blots of data in Fig. 1a of Wisp2 protein in different tissues from wt and Tg mice. (**b**) The full-length blot of data in Fig. 1bshowing Wisp2 protein in serum from wt and Tg mice on HFD. (**c**) The full -length blots of data in Fig. 4g of GLUT4 protein and ERK1/2 as loading control in

isolated mature sWAT from wt and Tg HFD mice. (**d)** The full-length blots of data in Supplementary Fig.1b of Wisp2 protein in peritoneal macrophages of wt and Tg mice. (**e**) The full length-blots of data in Supplementary Fig.3b of UCP1 and AKT protein in BAT from wt and Tg mice on HFD. (**f**) The full-length blots of data in Supplementary Fig.6a of SMAD2, Phospho-Smad2 and Actin protein as loading control in NIH C3H T101/2 cells.

Supplementary Tables

Supplementary Table 1. pQCT in femur and tibia of 23 weeks old mice on LFD or HFD

Supplementary Table 1	pQCT in femur and tibia of 23 weeks old mice on LFD or HFD					
**Femur**	**LFD wt**	**LFD Tg**	**P-value**	**HFD wt**	**HFD Tg**	**P-value**
Total BMD (mg/cm3)	474±23.2	518 ±14.9	0.056	472±11.4	498±16.2	NS
Trabecular BMD (45%) (mg/cm3)	285±24.1	332±17.8	NS	258±12.9	296±21.7	NS
Cortical CNT (mg/mm)	1.29±0.05	1.41±0.02	0.022	1.28±0.04	1.30±0.03	NS
Cortical DEN (mg/cm3)	1228±6.69	1237±4.65	NS	1221±5.97	1218±5.66	NS
Cortical bone area (mm2)	1.05±0.04	1.14±0.02	0.020	1.04±0.03	1.07±0.02	NS
Cortical THK (mm)	0.21±0.01	0.23±0.01	0.002	0.21±0.01	0.22±0.01	NS
**Tibia**	**LFD wt**	**LFD Tg**	**P-value**	**HFD wt**	**HFD Tg**	**P-value**
Total BMD (mg/cm3)	433±18.0	447± 13.5	0.049	447±11.0	453±13.3	NS
Trabecular BMD (45%) (mg/cm3)	271±18.1	272±15.0	NS	275±14.3	271±14.8	NS
Cortical CNT (mg/mm)	1.14±0.04	1.11±0.03	NS	1.09±0.03	1.11±0.03	NS
Cortical DEN (mg/cm3)	1167±7.97	1165±6.76	NS	1158±6.56	1161±5.85	NS
Cortical bone area (mm2)	0.98±0.03	0.95±0.02	NS	0.94±0.03	0.95±0.02	NS
Cortical THK (mm)	0.24±0.01	0.24±0.01	NS	0.23±0.01	0.23±0.01	NS

Abbreviations: wt, wild-type mice; Tg, transgenic mice; LFD, low-fat diet; HFD, high-fat diet; NS, non-significant.
Total BMD, Total bone mineral density; Cortical CNT, Cortical bone mineral content; Cortical DEN = Cortical bone density; Cortical THK= Cortical bone thickness.
The experimental data are presented as mean ± SEM. 2-way ANOVA was used. n ≥ 10/group.

Supplementary Table 2. mRNA expression in liver and gastrocnemius muscle

**Supplementary Table 2**	mRNA expression in liver and gastrocnemius						
**Liver**	**HFD wt**	**HFD Tg**	**P-value**	**Gastrocnemius**	**HFD wt**	**HFD Tg**	**P-value**
**Fatty acid transport/lipogenesis**				**Insulin signaling/action**			
Chrebpa	2.47±0.19	2.39±0.16	NS	*Glut4*	36.6±3.34	48.4±1.73	0.002
Chrebpb	9.98±2.13	5.61±1.23	0.067	*Insr*	1.65±0.16	1.47±0.07	NS
Elovl6	0.19±0.02	0.10±0.02	NS	*Irs1*	3.94±0.33	3.95±0.18	NS
Fasn	0.28±0.04	0.19±0.03	NS				
Ppara	3.44±1.15	3.67±1.53	NS				
Scd1	0.35±0.07	0.23±0.04	NS				

Abbreviations: wt, wild-type mice; Tg, transgenic mice; LFD, low-fat diet; HFD, high-fat diet; NS, non-significant. Chrebpa/b, Carbohydrate-responsive-element-binding protein alpha/beta; Elovl6, Elongation of long-chain fatty acids family member 6; Fasn, Fatty acid synthase; Ppara; Peroxisome proliferator-activated receptor alpha; Scd1, Stearoyl-CoA desaturase-1; Glut4, Glucose transporter type 4; Insr, Insulin receptor; Irs1, Insulin receptor substrate 1.
The experimental data are presented as relative quantitiy of mean ± SEM. Student's t-test was used, n ≥ 11/group.

Supplemental References:

1. Ray A, Dittel BN. Isolation of mouse peritoneal cavity cells. *J. Vis. Exp.* **35**, e1488, doi:10.3791/1488 (2010).

2. Chirgwin JM, Przybyla AE, MacDonald RJ, Rutter WJ. Isolation of biologically active ribonucleic acid from sources enriched in ribonuclease. *Biochemistry* **18**, 5294-5299 (1979).

3. Wu C*, et al.* BioGPS: an extensible and customizable portal for querying and organizing gene annotation resources. *Genome Biol* **10**, R130 (2009).

4. Su AI*, et al.* A gene atlas of the mouse and human protein-encoding transcriptomes. *Proc. Natl Acad. Sci. USA* **101**, 6062-6067 (2004).
